# SWS Brain-Wave Music May Improve the Quality of Sleep: An EEG Study

**DOI:** 10.3389/fnins.2020.00067

**Published:** 2020-02-11

**Authors:** Dongrui Gao, Siyu Long, Hua Yang, Yibo Cheng, Sijia Guo, Yue Yu, Tiejun Liu, Li Dong, Jing Lu, Dezhong Yao

**Affiliations:** ^1^School of Computer Science, Chengdu University of Information Technology, Chengdu, China; ^2^The Clinical Hospital of Chengdu Brain Science Institute, MOE Key Lab for Neuroinformation, University of Electronic Science and Technology of China, Chengdu, China; ^3^Center for Information in Biomedicine, School of Life Sciences and Technology, University of Electronic Science and Technology of China, Chengdu, China; ^4^Department of Composition, Sichuan Conservatory of Music, Chengdu, China

**Keywords:** sleep, brain-wave music, electroencephalography, neural plasticity, power spectra analysis

## Abstract

**Aim:**

This study investigated the neural mechanisms of brain-wave music on sleep quality.

**Background:**

Sleep disorders are a common health problem in our society and may result in fatigue, depression, and problems in daytime functioning. Previous studies have shown that brain-wave music generated from electroencephalography (EEG) signals could emotionally affect our nervous system and have positive effects on sleep. However, the neural mechanisms of brain-wave music on the quality of sleep need to be clarified.

**Methods:**

A total of 33 young participants were recruited and randomly divided into three groups. The participants listened to rapid eye movement (REM) brain-wave music (Group 1: 13 subjects), slow-wave sleep (SWS) brain-wave music (Group 2: 11 subjects), or white noise (WN) (Control Group: 9 subjects) for 20 min before bedtime for 6 days. EEG and other physiological signals were recorded by polysomnography.

**Results:**

We found that the sleep efficiency increased in the SWS group but decreased in REM and WN groups. The sleep efficiency in the SWS group was ameliorated [*t*(10) = −1.943, *p* = 0.076]. In the EEG power spectral density analysis, the delta power spectral density in the REM group and in the control group increased, while that in the SWS group decreased [*F*(2,31) = 7.909, *p* = 0.005]. In the network analysis, the functional connectivity (FC), assessed with Pearson correlation coefficients, showed that the connectivity strength decreased [*t*(10) = 1.969, *p* = 0.073] between the left frontal lobe (F3) and left parietal lobe (C3) in the SWS group. In addition, there was a negative correlation between the FC of the left frontal lobe and the left parietal lobe and sleep latency in the SWS group (*r* = −0.527, *p* = 0.064).

**Conclusion:**

Slow-wave sleep brain-wave music may have a positive effect on sleep quality, while REM brain-wave music or WN may not have a positive effect. Furthermore, better sleep quality might be caused by a decrease in the power spectral density of the delta band of EEG and an increase in the FC between the left frontal lobe and the left parietal lobe. SWS brain-wave music could be a safe and inexpensive method for clinical use if confirmed by more data.

## Introduction

Sleep disorders, affecting up to 30% of adults, are a common health problem in our society and may result in fatigue, depression, and problems in daytime functioning (Chang et al., 2012). Pharmacological treatment is helpful for people suffering from sleep disorders but also has side effects, and some of these individuals could possibly turn to psychological treatment.

As a non-pharmacological treatment, music can affect sleep disorders, as shown in a number of studies. Experiments with subjects listening to music before sleep have revealed that listening to soft music shortens the duration of deep sleep and prolongs the duration of deep sleep (Chang et al., 2012; Chen et al., 2014). In addition, subjects who listened to music had a longer sleep duration, greater sleep efficiency, shorter sleep latency, less sleep disturbance, and less daytime dysfunction as assessed by the Pittsburgh sleep quality index (PSQI) questionnaire (Tan, 2004). Similar results in an assessor-blinded randomized controlled trial (RCT) design showed a positive impact on sleep perception and quality of life (Jespersen et al., 2019). In electroencephalography (EEG) studies using time-frequency analysis methods, Kusumandari et al. (2018) demonstrated that music stimulation improved sleep quality.

Electroencephalography contains a wealth of information about brain activity. Scale-free brain-wave music, generated from EEG signals according to the power law of both EEG and music, possesses the characteristics of both music and EEG, and may contain physiological information that music alone may not (Wu et al., 2010, 2014; Lu et al., 2012). In recent years, brain-wave music has been shown to improve some clinical symptoms, such as pain (Levin, 1998). Levin’s (1998) work, which used a combination of behavioral data and power spectral density, showed that brain-wave music incorporates factors of music therapy and biological feedback (Huang et al., 2016). Brain-wave music has been applied in the treatment of orofacial pain, and the results showed that the brain-wave music and cognitive behavioral therapy (CBT) group had lower levels of pain perception than the control group. In addition, the brain-wave music group showed lower EEG complexity and slower waves (Zhuang et al., 2009). Brain-wave music can also provide us with a new way to examine alterations in brains across various populations. The brain-wave music of healthy subjects and epilepsy patients clearly revealed differences in the two brain states, in that the brain music from the epilepsy patients was composed of unusual variations (Yao et al., 2016). Classic studies have allowed us to further explore neural mechanisms. Sleep staging and the PSQI questionnaire have been used to evaluate sleep quality in previous studies, and the results of the behavioral data showed that brain-wave music has a positive effect. However, the neural activities underlying the improvement in the quality of sleep by brain-wave music still need to be clarified. Therefore, our motivation of this study is to uncover this neural mechanism described above.

As representative sleep stages, rapid eye movement (REM) sleep repairs advanced cognitive function, and N3 stage sleep, also called slow-wave sleep (SWS) or deep sleep, can relieve fatigue (Griessenberger et al., 2013). We generated two types of scale-free brain-wave music as music stimulation, one from the REM stage and the other from the SWS stage. Deep sleep can predict sleep satisfaction and is a representative indicator of sleep quality (Riedel and Lichstein, 1998), so for EEG analysis, we mainly analyzed the power spectrum of EEG during deep sleep, and explored the neural mechanisms of these two brain-wave music on sleep promotion from the perspective of EEG.

## Materials and Methods

### Participants

The study was implemented at a sleep center at the Clinical Hospital of Chengdu Brain Science Institute, University of Electronic Science and Technology of China (UESTC).

To observe the effect of brain-wave music on sleep, participants with a regular habit of staying up late were enrolled in the experiment. We recruited 36 right-handed subjects who had the sub-healthy sleep quality (PSQI scores should be between 4 and 8) from UESTC, and three of them gave up in midway through the experiment. The data of the remaining 33 participants (16 females; mean = 21.4 ± 5.6 years of age) were finally included in our experiment. All subjects gave informed consent for participation and received compensation.

### Music Stimulation

**FIGURE 1:**
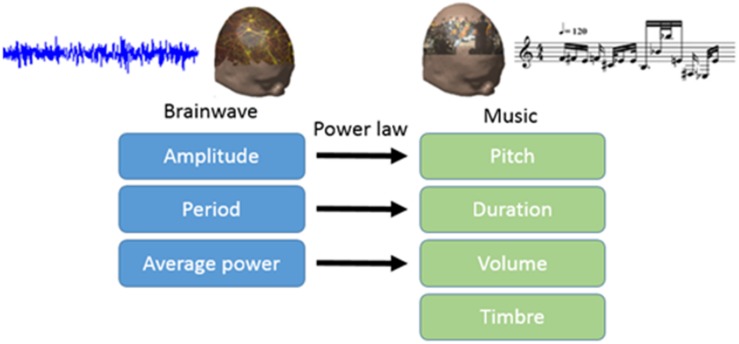
Paradigm for translating electroencephalography (EEG) into brain-wave music.

**FIGURE 2:**
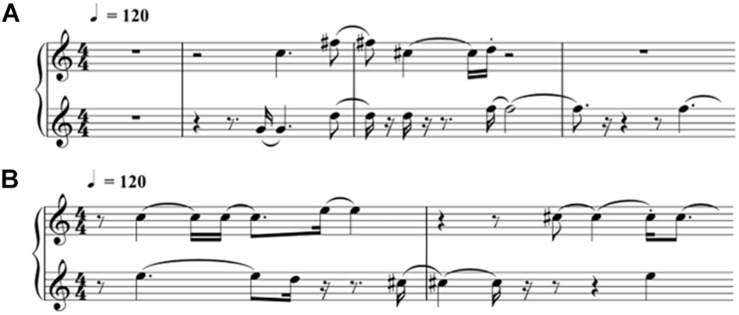
Illustration of the brain-wave music for the experiment [printed by Sibelius (2019)]. The notes from the REM brain-wave music at the beginning are shown in **(A)**, and the notes from the SWS brain-wave music at the beginning are shown in **(B)**.

In this study, we translated EEG into brain-wave music with the paradigm shown in Figure 1 (Wu et al., 2009; Lu et al., 2012). We used two pieces of brain-wave music in the experiment. One piece was REM brain-wave music, which was generated from EEG during the REM sleep. Another piece was SWS brain-wave music, which was generated from EEG during the SWS sleep. Some musical notes for each piece of music are shown in Figure 2.

### Electroencephalography Data Acquisition

**FIGURE 3:**
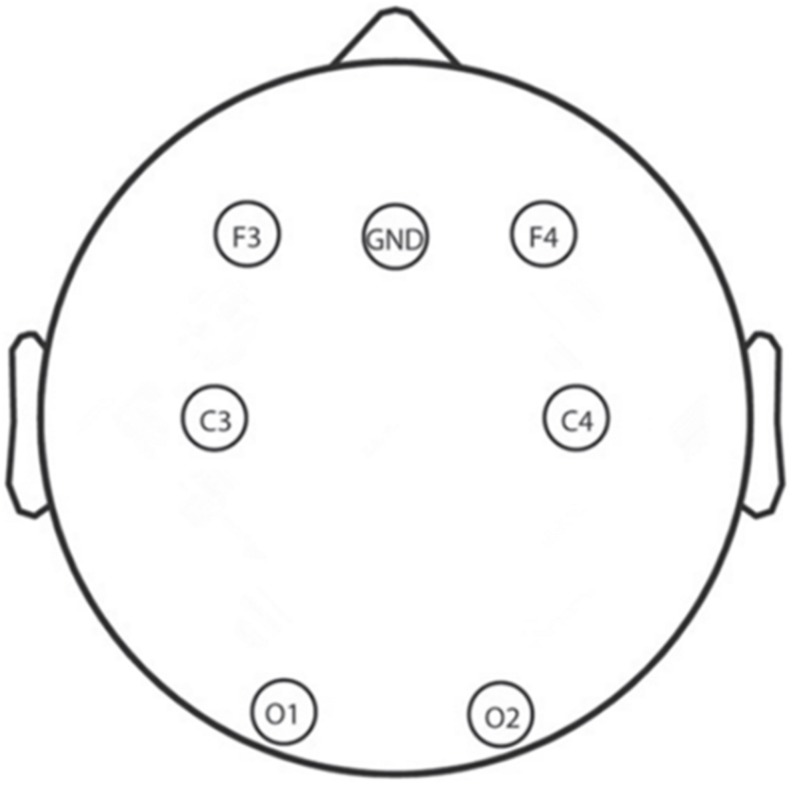
Electroencephalography electrode layout.

A total of 6 Ag/AgCl electrodes (F3, F4, C3, C4, O1, and O2) that obtain signals related to sleep and other physiological signals from 10 to 20 system were selected for EEG recording by using an Alice 5 LDx system (Philips Respironics, PA, United States; Lucey et al., 2016; Gozal et al., 2019). A montage of the six electrodes used in this study is shown in Figure 3; these electrodes were chosen to cover the main portion of the brain so that we could calculate the power spectral density and build the whole brain network to examine neural mechanisms. The bilateral mastoids were linked as the reference, and all other electrodes were kept below 10 kΩ. The EEG signals were sampled at 200 Hz which and filtered between 0.5 and 30 Hz with a bandpass filter.

### Experimental Procedures

**FIGURE 4:**
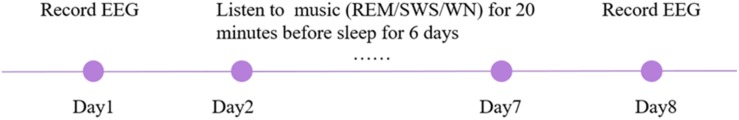
Illustration of the experimental procedure.

To avoid the effects of group differences in the initial state of sleep, participants were divided into three groups randomly. In reference to a compelling study about lullaby-accelerated falling asleep in children, the participants listened to REM brain-wave music (Group 1: 13 subjects, mean age = 21.69 ± 2.31), SWS brain-wave music (Group 2: 11 subjects, mean age = 21.77 ± 5.23), or white noise (WN; Control Group: 9 subjects, mean age = 19.78 ± 3.22 years) for 20 min before bedtime for 6 days (Patterson, 2011). EEG and other physiological signals were recorded by polysomnography on the first day and last day. The whole experiment lasted 8 days (Figure 4).

### Data Analysis

According to previous studies, the period of N3 stage in the proportion of total sleep time and sleep latency can predict sleep satisfaction and are representative indicators of sleep quality (Riedel and Lichstein, 1998). A sleep latency of less than 15 min is rated as an appropriate measure for indexing good sleep quality (Ohayon et al., 2017). Meanwhile, sleep efficiency is also correlated to sleep quality (Jankelowitz et al., 2005), and a sleep efficiency of more than 85% is judged as an appropriate indicator of good sleep quality (Buysse et al., 1991; Åkerstedt et al., 1994; Ohayon et al., 2017). Therefore, we chose these three indicators for our behavioral data analysis.

Sleep stage assessment in the first session was based on EEG, electro-oculography (EOG), electrocardiography (ECG), and electromyography (EMG), according to the American Academy of Sleep Medicine (AASM) criteria and the identified EEG signals of deep sleep. Sleep is divided into five stages: W, R, N1, N2, and N3 in the AASM criteria, and EEG is obtained in the deep sleep stage, where fatigue is effectively relieved (Danker-Hopfe et al., 2009). According to the AASM, the delta wave accounts for more than 20% of a frame during the N3 stage. Moreover, total sleep time, sleep efficiency, sleep latency, and percentage of time in each sleep stage were calculated (Suzuki et al., 2019). Yue Yu, a physician of sleep medicine, extracted the EEG signal either from the N3 stage alone or from eight sets of N2 data, which is similar to N3 (the delta wave accounts for more than 15% of a frame), as the deep sleep data when the participant lacked the N3 stage according to the AASM criteria.

Deep sleep EEG was preprocessed by the reference electrode standardization technique (REST) with zero reference (Yao, 2001; Yao et al., 2019) and 0.5–30 Hz bandpass filtering under the Webrain platform^1^. Consider that the delta band (0.5–4 Hz) is the dominant frequency of EEG at N3 stage and related to the quality of sleep (Danker-Hopfe et al., 2009), we calculated power spectral density and brain network connectivity in delta band after preprocessing. The results were made clear through correlations among the total sleep time, sleep efficiency, sleep latency, percentage of time in each sleep stage, and EEG data (Guevara and Corsi-Cabrera, 1996; Chennu et al., 2016; Comsa et al., 2019).

### Statistical Analysis

Both the time of data collection and the intervention used (REM, SWS, and WN) were factors. All EEG data processing was based on the EEG of N3 stage sleep or deep sleep. Comparison of the slight differences in the power spectral density among the three groups were assessed with ANOVA. *T*-tests were performed during the analysis.

### Strategy for Removing Outliers

Data from subjects who were not asleep or did not have N2 and N3 stages, as assessed by the sleep recording data, were considered outliers according to the AASM criteria. To obtain clear EEG data, we carefully eliminated some of the data obtained through bad channels.

## Results

**FIGURE 5:**
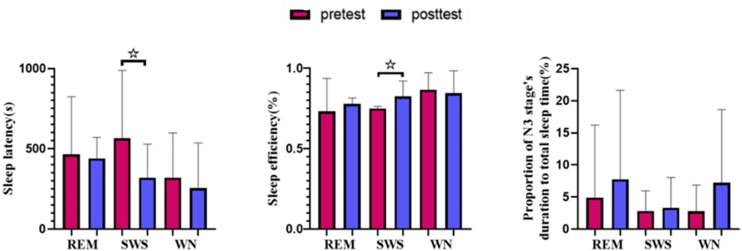
Sleep variables from polysomnography (PSG). The sleep latency in the SWS group decreased significantly [*t*(10) = 2.441, *p* = 0.031] after listening to music, and the sleep efficiency in the SWS group increased significantly [*t*(10) = −1.943, *p* = 0.076]. * means there is a significant difference or a marginally significant difference between two groups.

We found that the sleep latency in the SWS group decreased by 38.45% [*t*(10) = 2.441, *p* = 0.031] after listening to music. Although the sleep latency in the WN group and REM group subjects also decreased after the intervention, the differences were not significant. The sleep efficiency ($\mathrm{sleep}\,\mathrm{efficiency}=\,\frac{s\,l\,e\,e\,p\,\,t\,i\,m\,e}{b\,e\,d\,\,t\,i\,m\,e}$) in the SWS group increased by 3.98% [*t*(10) = −1.943, *p* = 0.076], while in the other two groups, the sleep efficiency decreased. The percentage of sleep time spent in stage N3 increased in all three groups but not to a statistically significant degree (Figure 5).

**FIGURE 6:**
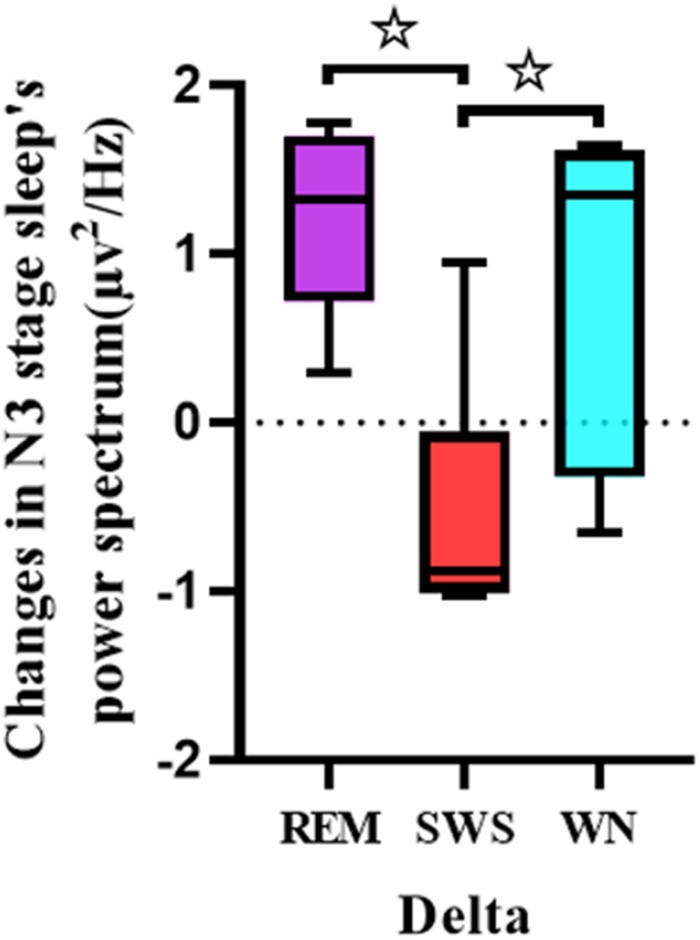
Comparison of each group’s whole brain power spectral density in the delta band of the EEG during the deep sleep stage (posttest – pretest). For the delta band of the EEG, there was a significant difference between the REM group and the SWS group (*p* = 0.005) and between the SWS group and the WN group (*p* = 0.024). * means there is a significant difference or a marginally significant difference between two groups.

One-way ANOVA was used to analyze the differences between the pretest and posttest whole brain power spectral density in the delta band of the deep sleep EEG among the REM, SWS, and WN groups (Figure 6). The SWS group’s whole brain power spectral density decreased, while the other two groups showed increases in the delta band [*F*(2,31) = 7.909, *p* = 0.005].

**FIGURE 7:**
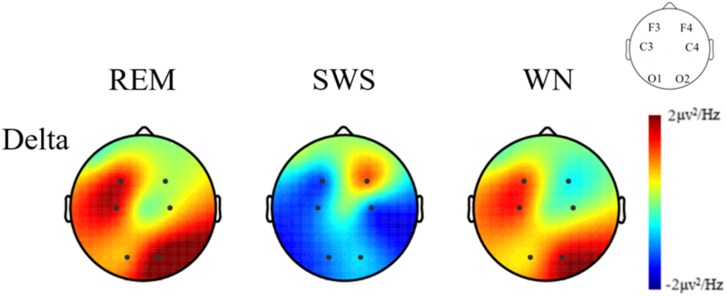
The EEG power spectral density topographic maps of the deep sleep stage for the REM, SWS, and WN groups in the delta band, respectively (posttest – pretest). In the REM group and the WN group, the power spectral density at the F4 and C4 channels did not change after music listening. The power spectral density at the O1, C3, F3, and O2 channels all increased after music listening, of which the O1 channel increased the least and O2 increased the most. In the SWS group, the power spectral density at the F4 channel did not change, and the power spectral density at the F3, C3, C4, O1, and O2 channels decreased after the experiment, with the C4 channel decreasing the most. The power spectral density increased in F4 but decreased in other channels in the SWS group.

We further analyzed the EEG power spectral density topographic maps in the delta band of the deep sleep stage for the three groups. The power spectral density for the whole brain in the delta band increased in the REM and WN groups, while the completely opposite effect was observed in the SWS group (Figure 7).

**FIGURE 8:**
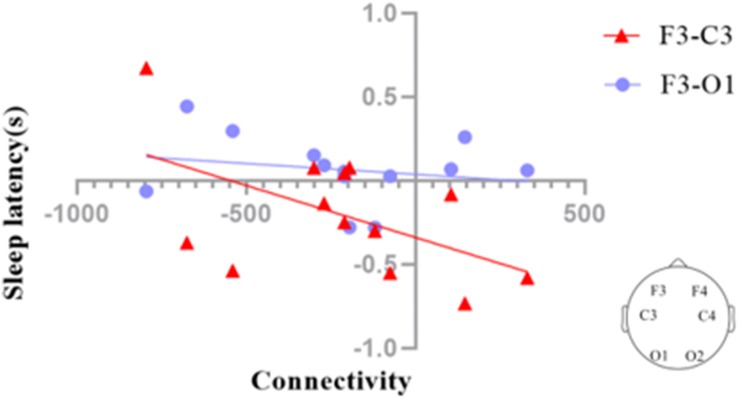
Correlation coefficients between the EEG connectivities of the deep sleep stage and sleep latency in the SWS group (posttest – pretest). The connectivity between F3 and C3 (F3–C3) during deep sleep was marginally significantly correlated (*p* = 0.064) with sleep latency (red triangles), while the connectivity between F3 and O1 (F3–O1) during deep sleep was not significantly correlated with sleep latency (blue dots).

We investigated the functional connectivity (FC) of different regions of the brain via graph theory, which consists of nodes and edges. In our analysis, the scalp electrodes were defined as the nodes, and the Pearson correlation coefficients between nodes were defined as the edges. We also calculated the correlation coefficients between the F3–C3 and F3–O1 connectivities and sleep latency (Figure 8). The results suggested that sleep latency was inversely correlated with F3–C3 connectivity (*r* = −0.527, *p* = 0.064), meaning that a larger increase in the connectivity for F3–C3 could lead to a larger decrease in sleep latency.

## Discussion

Some previous studies have shown that listening to the subjects’ own brain-wave music could improve the quality of sleep (Levin, 1998), while another study found that listening to healthy subjects’ brain-wave music might be more useful (Yao et al., 2016). There is no agreement on what kinds of brain-wave music can improve the quality of sleep. REM sleep could repair advanced cognitive function, and SWS sleep could relieve fatigue (Griessenberger et al., 2013). Therefore, we chose these two kinds of EEG in different periods of sleep and generated the brain-wave music from them. As a result, our study found that SWS brain-wave music could improve sleep quality but REM brain-wave music could not. In order to understand its mechanism, we did further analysis on both behavioral and EEG data.

Sleep latency can be interpreted as a sense of sleepiness before going to bed and is a very important part of sleep quality (Chen et al., 2014). Music intervention before bedtime may facilitate relaxation as a person falls asleep (Steelman, 1990; Updike, 1990; White, 1992). It was found that listening to SWS brain-wave music at bedtime can shorten sleep latency, which is consistent with Levin’s (1998) experimental results. In another study, listening to sedating music did not significantly alter sleep latency (Higuchi et al., 2005). Therefore, SWS brain-wave music may have a better effect with regard to relaxation than sedating music.

Normally, the delta band brain-wave is generated during sleep and relaxed conditions (Kumarahirwal and Londhe, 2013). A lower power in low-frequency band EEG indicates better sleep, especially deep sleep (Svetnik et al., 2017). It was found that the power spectral density in the SWS group decreased in the delta band, which is consistent with experimental results in subjects using benzodiazepines and zolpidem (Monti et al., 2000; Bastien et al., 2003). A study also found that with increasing age, the activity in the delta band decreases in power, which may be related to an attenuation of homeostatic sleep pressure and to an increase in cortical activation during sleep (Carrier et al., 2001). Therefore, the decrease in the delta brain waves in our study may have been indicative of an elevated sleep propensity and a relief from homeostatic sleep pressure in the SWS group (Esposito and Carotenuto, 2014). The power spectral density in the SWS group increased, while that in the other two groups decreased in the delta band (posttest – pretest). There were significant differences between the SWS and REM groups and between the SWS and WN groups. We could conclude that SWS brain-wave music had a positive effect.

However, in the delta band, the power spectral density of the REM and WN groups increased, and there was no significant difference between the two groups, indicating that REM brain-wave music and WN have similar effects on the EEG power spectrum. In Alexander’s study, he found that the EEG power density in the low-frequency range (delta band) was an indicator of a progressively decreasing process during sleep (Borbély et al., 1981). It seemed that with the deepening of sleep, the power of delta frequency band decreased simultaneously. In our experiment, we found that after REM brain-wave music or WN listening, the power of delta band increased during sleep (posttest – pretest), and suggesting that these two kinds of music may have a negative effect on the deepening of sleep.

Overnight sleep deprivation leads to reduced activation of the frontal and parietal lobes (Chee and Tan, 2010). A meta-analysis showed brain activation in the right prefrontal cortex and medial frontal cortex was significantly reduced following sleep deprivation compared to rested wakefulness and that the activation in the frontoparietal attention network was reduced following acute total sleep deprivation compared to normal resting (Ma et al., 2015). These findings suggested that the decrease in this connectivity may be related to increased sleepiness and a greater likelihood of falling asleep. Chee et al. (2006) found that activation of the left frontal parietal lobe after normal sleep was negatively correlated with the performance accuracy decreases observed between normal sleep conditions and sleep deprivation over 24 h. In another study, Zou et al. (2018) found that the FC in the left frontoparietal network showed strong a correlation with REM sleep percentage. It appears that the activity of the left frontal and parietal lobes is highly correlated with various aspects of sleep. In our experiment, we found that the connectivity of the left frontal (F3) and parietal (C3) lobes was linked with sleep latency, so we speculate that the connectivity of the left frontal and parietal lobes may affect sleep latency.

This study has some limitations. First, insomniac patients should be recruited to determine the therapeutic effect of SWS brain-wave music on sleep in the future. Secondly, only two representative types of brain-wave music were selected in this experiment. Whether other types of music can promote sleep and the neural mechanisms remains to be further studied. Finally, further study should also consider the different effects on improving sleep quality between the brain-wave music and other types of music, such as classical music.

## Conclusion

This was an exploratory study on how SWS brain-wave music affects deep sleep. We suggest that SWS brain-wave music can decrease the delta band EEG power spectral density, shorten sleep latency and significantly correlate the F3–C3 connectivity, and sleep latency to improve sleep quality. However, REM brain-wave music and WN may not improve the quality of sleep.

## Data Availability Statement

The datasets generated for this study are available on request to the corresponding author.

## Ethics Statement

The studies involving human participants were reviewed and approved by the Ethics Committee of the School of Life Science and Technology at the University of Electronic Science and Technology of China. The patients/participants provided their written informed consent to participate in this study.

## Author Contributions

DG, SL, HY, YC, JL, and DY designed the experiments. DG, SL, YC, and YY performed the experiments and collected the data. DG, SL, YC, SG, TL, LD, and JL analyzed the data. SL, YC, JL, and DY interpreted the results of experiments. All authors wrote and revised the manuscript and approved the submitted version.

## Conflict of Interest

The authors declare that the research was conducted in the absence of any commercial or financial relationships that could be construed as a potential conflict of interest.

## Footnotes

1 http://webrain.uestc.edu.cn/
